# Community risk of environmental-borne cystic echinococcosis transmission in South America: Results from the multistep cross-sectional and case-control PERITAS study

**DOI:** 10.1371/journal.pntd.0013382

**Published:** 2025-08-06

**Authors:** Gerardo Acosta-Jamett, Francesca Tamarozzi, Natalia Castro, Saul J. Santivanez, Raul Enriquez Laurente, Cristina Mazzi, Cristian A. Alvarez-Rojas, Adriano Casulli

**Affiliations:** 1 Instituto de Medicina Preventiva Veterinaria, Universidad Austral de Chile, Valdivia, Chile; 2 Center for Disease Surveillance and Evolution of Infectious Diseases, Facultad de Ciencias Veterinarias, Universidad Austral de Chile, Valdivia, Chile; 3 Department of Infectious-Tropical Diseases and Microbiology, IRCCS Sacro Cuore Don Calabria Hospital, Negrar di Valpolicella, Verona, Italy; 4 Research Department, Universidad Continental, Huancayo, Peru; 5 Center for Global Health, Universidad Peruana Cayetano Heredia, Lima, Peru; 6 Clinical Research Unit, IRCCS Sacro Cuore Don Calabria Hospital, Negrar di Valpolicella, Verona, Italy; 7 Escuela de Medicina Veterinaria, Facultad de Agronomía y Sistemas Naturales, Facultad de Ciencias Biológicas y Facultad de Medicina, Pontificia Universidad Católica de Chile, Santiago, Chile; 8 European Union Reference Laboratory for Parasites (EURL-P), Department of Infectious Diseases Istituto Superiore di Sanità, Rome, Italy; 9 WHO Collaborating Centre for the Epidemiology, Detection and Control of Cystic and Alveolar Echinococcosis (One Health), Department of Infectious Diseases, Istituto Superiore di Sanità, Rome, Italy; Research Center for Hydatid Disease in Iran Kerman University of Medical Sciences, IRAN, ISLAMIC REPUBLIC OF

## Abstract

**Background:**

Cystic echinococcosis (CE) is mainly described as a food/waterborne zoonosis. However, evidence about matrices contamination is scarce. Identifying main transmission routes could optimize health messages aiming to prevent ingestion of parasite eggs. We evaluated *Echinococcus granulosus* contamination of matrices in two areas of Chile and Peru.

**Methodology/principal findings:**

In stage 1, areas with high active CE prevalence were identified through cross-sectional ultrasound surveys. Stage 2 was a case-control study encompassing matrices sampling in public places and households with and without CE cases in these areas, followed by (stage 3), matrices processing by sequential sieving and *E. granulosus* detection by PCR. Bayesian multilevel mixed-effects logistic regression analysis was used to identify factors associated with risk of contamination. In households, soil (19%-42%); dogs’ fur (10%-30%); shoes’ soles (5%-33%); and dogs’ feces (0–50%) were highly contaminated. In public areas, ~ 30% of fecal and soil samples were contaminated. Overall, matrices from public areas were more contaminated than those from households. When examining households, there was no difference in risk of contamination according to presence of CE cases, while CE-free households had lower risk when considering households and public areas. There was no difference in risk of contamination according to matrix. Vegetables were PCR-negative.

**Conclusions/significance:**

Results suggest the need for a paradigm-shift towards considering CE an environmental-borne infection with a “community risk” to which people are exposed.

## Authors summary

Cystic echinococcosis (CE) is a parasitic zoonosis transmitted between dogs and livestock, while humans are accidental dead-end hosts, becoming infected through ingestion of viable parasite eggs shed with the feces of infected dogs. Since hygiene measures aiming to prevent the ingestion of eggs by humans constitute the only human-targeted primary prevention activities, identifying the main routes of transmission in endemic areas could optimize targeted, pragmatic health education messages. CE is mainly described as a food/waterborne zoonosis or deriving from “contact with dogs”. However, evidence about parasite contamination of different materials is scarce and published data suggest that environmental contamination could be one of the main sources of infection for humans. We evaluated parasite contamination of different materials in two villages of Chile and Peru, comparing contamination of different materials in households with and without active CE cases diagnosed during an ultrasound-based population study. Materials collected in public areas were also analysed for contamination. We found that materials from public areas were more contaminated than those from households and there was no difference in risk of contamination according to materials. These results suggest the need for considering CE an environmental-borne infection with a “community risk” to which people are exposed.

## Introduction

Cystic echinococcosis (CE) is the parasitic zoonosis caused by infection with the larval stage of the cestode *Echinococcus granulosus sensu lato* species complex. It is estimated that over 1 million people worldwide are infected, with over 800,000 DALYs lost and US$ 3 billion spent yearly for treating cases and losses to the livestock industry [1]. Despite these figures, which are likely underestimated due to underreporting and misreporting of data, and despite being listed among the Neglected Tropical Diseases (NTD) targeted for control, CE control programmes are not widely implemented [2,3].

The parasite life cycle develops mainly in a domestic rural environment between canids (most commonly domestic dogs involved in herding or free-roaming dogs) and livestock (most commonly sheep) [4,5]. Dogs are definitive hosts harbouring the adult parasites in the small intestine; despite adult worms being relatively short-lived (10–12 months [6]) and most eggs being excreted within a couple of months from infection [7], readily infective parasite eggs shed with the infected dog faeces can survive and contaminate the environment for many months in optimal temperature and humidity conditions [8]. Ungulates are intermediate hosts becoming infected through ingestion of viable parasite eggs in pastures; in the intermediate hosts, the parasite develops into the metacestode (larval) stage, in the form of fluid-filled cysts (echinococcal cysts, commonly referred to as “hydatid cysts”) in internal organs, mainly liver and lungs. Transmission to the definitive host is achieved when dogs feed on ungulates’ offal containing fertile echinococcal cysts (i.e., containing protoscoleces), which can be promoted by human behaviour in case of domestic, “informal” slaughtering.

Humans are accidental dead-end hosts, becoming infected through ingestion of viable parasite eggs; when infection is successful, echinococcal cysts develop mainly in the liver (about 70%) and lungs (about 20%), although all organs and tissues can be infected [9]. The course of infection is slow (months to decades) and symptoms, whenever they develop, are largely non-specific and caused by mass-effect on neighbouring organs or tissues, or intervening complications (i.e., rupture or bacterial superinfection). The infection episode (i.e., the precise episode of eggs ingestion followed by successful infection) does not produce evident manifestations, and the uneven growth rate of the metacestode over time does not allow dating the age of the infection [10]. Therefore, although it is well-known that human infection occurs via the faecal-oral route (from dog feces to oral ingestion of eggs by humans), it is virtually impossible to trace back when the infection actually occurred and through what contaminated matrix.

CE is commonly described as a food/waterborne infection or an infection deriving from “contact with dogs”. As a foodborne infection, CE is actually ranked the second most significant parasitic food-borne diseases worldwide by the Food and Agriculture Organization (FAO) and the WHO [11], and classic primary prevention recommendations, in both scientific literature and lay divulgation material, include “washing raw vegetables before consumption”, “avoiding direct contact with dogs and “washing hands after touching dogs”. These transmission routes (ingestion of contaminated raw vegetables, hand-to-mouth or dog-to-mouth transfer) are biologically plausible. However, evidence about real contamination by infective *E. granulosus* eggs of vegetables and water for human consumption and of other matrices (soil, dog fur, etc.) is difficult to ascertain due to the unavailability of an easy to use eggs viability test, and, more in general, overall data about (any) *E. granulosus* eggs contamination in different matrices and attributable fractions (i.e., the proportion of disease attributed to a particular route/risk factor) are scarce [12–14]. A general appraisal of the literature seems indicating that environmental contamination could be one of the main sources of infection for humans; furthermore, socio-cultural and area-specific circumstances, including lack of access to clean water for hand washing, most likely influence transmission risk to humans [13].

Since humans are dead-end hosts for *E. granulosus*, treatment of patients does not impact infection transmission, as opposed to many other parasitic NTDs such as soil transmitted helminthiases. While control measures for CE aiming to interrupt transmission can therefore only be based on animal-targeted interventions (dog’s deworming, sheep vaccination, culling of aged sheep, organs’ disposal after slaughter impeding dogs’ access) [2], measures aiming to prevent the actual ingestion of eggs by humans are the only applicable human-targeted primary prevention activities. Identifying and quantifying the site-specific main routes of transmission in endemic areas could optimize targeted, pragmatic health education messages and campaigns. To this end, studies on matrices contamination complemented by epidemiological investigation on site-specific at-risk habits are pivotal, together with social-science investigations on habits putting different communities at different risks of contact with contaminated matrices.

In this study, we aimed to evaluate *E. granulosus* contamination of households- and public locations-derived matrices in two highly endemic areas of Chile and Peru, identified through ultrasound-based CE screening studies performed during the project PERITAS (Molecular epidemiological studies on pathways of transmission and long lasting capacity building to prevent cystic echinococcosis infection) [15,16].

## Methods

### Ethics statement

PERITAS project activities in Chile and Peru were approved by the Ethics Committees of the Faculty of Medicine, Universidad Católica del Norte, Coquimbo, in Chile (CECFAMED-UCN N38/2019), and of the Universidad Peruana Cayetano Heredia, Lima, in Peru (CONSTANCIA 440-14-19). All participants were asked to sign a written informed consent and parental/guardian consent for the inclusion of children.

The study is reported according to STROBE (Strengthening the Reporting of Observational Studies in Epidemiology) guidelines for reporting observational studies (S1 Checklist).

### PERITAS study design

**Fig 1:**
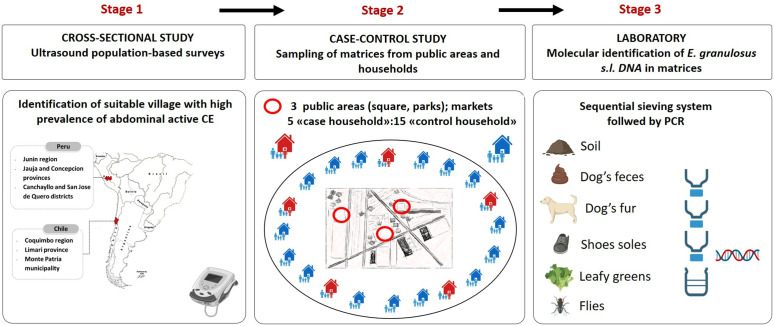
Stepwise design of the PERITAS study. The image contains CC-BY 4.0 licensed elements created in mapswire.com (https://mapswire.com/maps/south-america-political-maps/) and BioRender (https://BioRender.com/p5sbj2d) and assembled in PowerPoint (Microsoft Corporation).

PERITAS was conceived as a stepwise multidisciplinary study involving three stages (Fig 1). Stage 1 included an ultrasound population-based screening carried out in target areas in Argentina, Chile and Peru, aiming to estimate the prevalence of human CE and to identify areas/villages with both households (with and without CE cases) and public areas (e.g., village’s squares, parks, outdoor markets) attended by the inhabitants, where to carry out the environmental sampling. Stage 2 was a case-control study encompassing sampling of different matrices in the areas selected in stage 1, i.e., in households with (“case households”) and without identified CE cases (“control households”) and in public areas. Finally, Stage 3 aimed to detect *E. granulosus s. l.* DNA in the sampled matrices, to evaluate and compare contamination between areas and shed light on the pathways of infection transmission to humans. Stage 2 and stage 3 were carried out only in Chile and Peru; Argentina took part only in the ultrasound screening stage [17] but not in the matrices contamination assessment due to administrative and financial restrictions.

### Stage 1: Cross-sectional population-based ultrasound surveys for CE and identification of areas where to conduct the environmental sampling

The ultrasound-based studies were carried out in 2019 in the municipality of Monte Patria of the Limarí province, Coquimbo region, Chile [15], and in the districts of Canchayllo (Jauja province) and San José de Quero (Concepción province), both located in the Junín region of the Central Highlands in Peru. For the purpose of this study, active CE stages were those with liquid components, i.e., CE1, CE2, CE3a and CE3b according to the WHO-IWGE classification [18]. High prevalence of CE with active cyst stages was used to choose sampling areas assuming that an active CE cyst would reflect recent and ongoing transmission and arguably derive from a more recent infection than an inactive one [10].

### Stage 2: Case-control village-based environmental sampling of matrices

A general case-control matrices sampling strategy was applied. In each area, the village where to carry out the environmental sampling was identified based on the presence of high prevalence of CE with active cyst stages in the ultrasound surveys and the presence of at least five households with participants with abdominal active CE. In each selected area, five households with at least one CE case with active cysts detected in the ultrasound surveys (“case households”) and 15 (1:3 ratio) households with no CE cases in the ultrasound surveys (“control households”) were selected for matrices sampling. Care was taken to select households with similar environmental conditions in terms of occupation, dog ownership, living setting (e.g., rural setting, house structure) and, whenever possible, where all the family was screened by ultrasound. In the selected households, the following matrices sampling was envisaged: 1) Shoes: one adhesive tape per shoe sole of two shoes pairs from the same person; each adhesive tape was applied twice in two different areas of each sole and then stuck onto a glass slide. 2) Owned dogs’ feces: one rectal swab or 10 ml feces of each household’s dog; in case there was no possibility to collect feces directly from rectal sampling of each dog, one fresh fecal sample per number of household’s dogs was collected from the ground of the household’s premise. 3) Owned dogs’ fur: for each dog, one adhesive tape applied in four areas of the muzzle, and one applied in four areas of the perineum; after application on the fur, each tape was stuck onto a glass slide. 4) Soil: surface soil collected at the main house entrance, at the main entrance to the backyard from the public road, and in one area where the dogs are housed and/or tied. In each location within the household, approximately 300 g of soil were collected. For the collection of soil samples, a plastic bag changed after each sampling was used to cover the shovel, to avoid cross-contamination between sampling sites. 5) Up to three leafy green vegetables (300 grams) intended for raw consumption (e.g., lettuce heads) collected from each household, regardless of whether they were self-grown or purchased. The origin of the vegetables was asked to the household’s occupant and recorded.

In addition to households, three public areas (e.g., squares, parks) were identified in the village for the sampling of soil and dogs’ feces; one or more indoor markets or groceries were also identified for sampling of vegetables. Nine soil samples were envisaged to be collected from each public area using a systematic unaligned sampling strategy. Briefly, each public area was divided into nine sectors of equal surface area, and 300 g soil was collected in each sector, as described above. In case of asphalted areas, a dustpan and brush were used to collect the surface materials from 1 m^2^ surface. From the same public areas, dog’s faecal samples were collected using a walking path transect sampling strategy. Briefly, up to nine faecal samples were collected when encountered while walking along the border of the area; if possible, at least one sample per side of an ideal squared area was collected. Up to 40 heads of leafy green vegetables for raw consumption were envisaged to be collected from a village market, if present. Finally, one commercial flypaper trap was envisaged to be placed in each public area for three hours in the morning.

Each sample was collected in individual labelled and sealed plastic container and preserved at 4°C in the field and during transportation to the lab. Samples were frozen for 10 days at -80°C and then kept at -20°C until analysis.

### Stage 3: Molecular detection of *Echinococcus granulosus* DNA

While the study team of each country performed the matrices sampling following standard operating procedures, molecular analyses were centralized in Chile to decrease inter-laboratory variability. However, a common training on pre-analytical handling of samples and PCR standardization was organized to foster capacity building.

A sequential sieving system initially developed for the concentration of *Echinococcus/Taenia* eggs from carnivore faeces [19] and vegetables [20]. In the former case, we followed the methodology by Guggisberg et al. [20], in which the external leaves of lettuce (200 g approximately) was thoroughly washed with 500ml Twenn20 1% solution. After shaking in a sealable plastic bag, the content was passed by the sieving method. These techniques were adapted to be used with soil, flies, and tapes from soles and dog’s hair. Tape samples were first washed with 0.5% Tween 20, while flies were crushed and rinsed with the same solution; after vigorous shaking, both sample types were processed using the sieving system. Parasite eggs, if present, were concentrated based on their size in nylon filters with pores measuring 105, 40 and 21 µm as described in [20]. The sediment collected from each processed sample was mechanically disrupted using approximately 50 µL of 0.1 mm zirconia/silica beads (Biospec Products). The tubes were then agitated in a cell disruptor (MiniBeadbeater-8; Biospec Products) at 3200 g for 90 seconds. DNA was subsequently isolated using the QIAamp Fast DNA Stool Mini Kit (QIAGEN) following the manufacturer’s instructions.

The DNA samples were then used as a template for the detection of *Echinococcus* sp. by PCR according to the protocol of Stefanić et al. [21]. A multiplex PCR described by Trachsel et al. [22] was used to differentiate *Echinococcus* sp. from *Taenia* sp. Ten samples with stronger gel bands were selected to be Sanger sequenced to confirm the correct detection of *Echinococcus* spp. PCR results were reported as positive or negative.

### Statistical analysis

Due to the absence of previous data on contamination of environmental matrices by *E. granulosus* eggs, sample size calculations were based on estimation of prevalence of active abdominal CE cysts in the investigated villages for Stage 1 [15]. Based on a desired precision of 1% and a confidence level of 95%, and a maximum expected prevalence of active abdominal CE of 4%, the minimum US screening sample size was n = 374 in villages of 500 inhabitants, n = 597 in villages of 1000 inhabitants, and n = 850 in villages of 2000 inhabitants. For Stage 2 and 3, the sample size was based on available resources and capacity. Continuous variables were summarized using median and interquartile ranges (IQR), while discrete variables were summarized by absolute and percentage frequencies. The McNemar test was used to compare the frequency of contamination between dogs’ muzzle fur, perianal fur, and rectal swabs/feces. All other frequencies were compared using Fisher’s exact test. To identify the factors associated with contamination (i.e., location and matrices), we used a multilevel mixed-effects logistic regression analysis within a Bayesian framework, treating household/public area codes as the random effect. Bayesian analysis allows for the integration of additional external information into the model through prior distributions, which can enhance both the precision and credibility of estimated effect sizes. For the regression coefficients, we applied minimally informative priors following a normal distribution, while the variance was estimated using a Student-t (3, 0, 2.5) distribution.

The analysis was conducted with the Brms package [23], which uses the Hamiltonian Monte-Carlo (HMC) algorithm and its No-U-Turn Sampler (NUTS) variant within Stan [24]. For posterior distribution estimation, we ran 3,000 iterations with 1,000 used for warm-up, across 4 separate chains with random initial values, using 2 computational cores, and an adapt delta of 0.95.

As HMC analyses can be unreliable until the Markov chain reaches its stationary distribution, we assessed convergence by tracking the Rhat statistic and monitored effective sample sizes using both Bulk ESS and Tail ESS [25,26], ensuring that results met the necessary diagnostic criteria for reliability.

## Results

### Stage 1: Cross-sectional population-based ultrasound surveys for CE and identification of areas where to conduct the environmental sampling

In Chile, a total of 2,439 people were screened by ultrasound from 13 localities in Limarí province, Coquimbo region, finding a 1.6% mean prevalence of CE, with 51% of CE cysts being in active stages [15]. In Peru, a total of 1,181 people screened by ultrasound from 12 localities in Junin and Huancavelica regions, finding a 3.7% mean prevalence of CE, with 56% of CE cysts being in active stages.

Among screened localities, high prevalence of CE with active cyst stages and needed number of case-households, control-households, and public areas were identified in Tulahuen and Canchayllo from Chile and Peru, respectively.

In Chile, matrices sampling was carried out in Tulahuen, a rural area of 935 inhabitants in the municipality of Monte Patria of the Limarí province, Coquimbo region [15]. In this area, the ultrasound-based screening performed in 2019 found a CE prevalence of 4.5%, with 2.5% of participants having CE in active stages [15]. Five “case households” and 15 “control households” were selected. The median number of household’s members were 3 (range 2–6) in the “control households” and 2 (range 2–5) in the “case households”. The number of family members having received abdominal ultrasound was 2 (range 1–3) in the “control houses” and 2 (range 2–2) in the “case households”. The percentage of household members in “control houses” who did not undergo ultrasound ranged from 0-83% (median 50%).

In Peru matrices were collected from Canchayllo, a rural village located in Junin region, in the Peruvian Central Highlands. Of the 768 inhabitants of Canchayllo (data from Instituto Nacional de Estadistica e Informatica), 236 were evaluated by ultrasound in this study. A prevalence of CE of 9,3% was found, and 50% of them had at least one active cyst. Five “case households” and 15 “control households” could be selected. The median number of household’s members were 3 (range 1–6) in the “control households” and 4 (range 2–7) in the “case households”; the number of family members having received abdominal ultrasound was 2 (range 1–4) in the “control houses” and 2 (range 1–5) in the “case households”. The percentage of household members in “control houses” who did not undergo ultrasound ranged from 0-83% (median 25%).

### Stage 2: Case-control village-based environmental sampling of matrices

Original data are available in S1 Dataset.

In Tulahuen, Chile, five “case households”, 15 “control households”, and three public areas (two squares and one public playground) were selected. Two grocery stores that sold vegetables were also identified. Three (15%) households at the time of sampling did not own dogs, notably two of them being “case households”; the other households had a median of two dogs per household (range 1–7), for a total of 45 dogs. Matrices sampling in the households was carried out per protocol, with the exception of i) five (11.1%) dogs (four from “control households” and one from a “case household”) for which it was not possible to obtain all three samples (muzzle tape, perianal tape, and faeces/rectal swab); ii) soil, which could be collected from all three household’s locations in only 11/20 (55%) households; and iii) vegetables, which could be collected only from 9/20 (45%) households, in five cases of which deriving from own kitchen garden. In the three public areas, nine soil and nine faecal samples per area were collected with the exception of one area from which seven faecal samples were collected. Vegetables could be collected from two markets (8 samples in one market and two samples in the other), while flies could not be collected.

In Canchayllo, Peru, five “case households”, 15 “control households”, and three squares in public areas were selected. No market/grocery store could be identified, therefore no vegetables on sell could be sampled. All households had dogs, median two dogs per household (range 1–4), for a total of 35 dogs. In the households, only one pair of shoes per household could be sampled; furthermore, for four (11.4%) dogs (two from “control households” and two from “case households”) it was not possible to obtain all three samples (muzzle tape, perianal tape, and faeces/rectal swab). Soil could be collected from all three household’s locations in only 5/19 (26.3%) households, and no vegetables could be collected from households. In the three public areas, flies and nine soil samples per area were collected while two, seven and eight faecal samples could be collected in each area respectively.

### Stage 3: Molecular detection of *Echinococcus* DNA

When comparing by Fisher’s Exact test the proportion of PCR positivity for *E. granulosus* DNA (thereafter referred to as “contamination”) between matrices sampled in the “case households” and “control households” (Table 1), no differences were found for “dogs’ fur” and “shoe soles”, which were contaminated in up to one-third of samples. Dogs’ faeces/rectal swabs were significantly more contaminated in “case households” compared to “control households” in Chile (p = 0.041), while in Peru no PCR positivity was obtained in these matrices. In both areas, Tulahuen (Chile) and Canchayllo (Peru), a high level of contamination was found in households’ soil, reaching up to 41% in “case households” and 29% in “control households” in Peru (p = 0.022), and around 20% in both households’ groups in Chile. Vegetables were collected only in Chile’s households and grocery stores and were all PCR-negative for *E. granulosus* DNA. In both villages, there were no significant differences between contamination of soil from different household’s collection areas (main house entrance, backyard’s entrance, dogs’ housing area). As for contamination of dog’s fur in the muzzle and perianal areas, in dogs in Tulahuen the proportions of contamination were 23.8% and 35.0%, respectively, while in Peru 0.0% and 21.2%, respectively, with no significant differences between the fur’s sampling areas. Interestingly, in both villages, rectal swabs were less contaminated than fur’s tape in sampled dogs.

**Table 1:** Proportions of PCR positivity for *Echinococcus granulosus* DNA in matrices which could be collected at household’s level in Tulahuen (Chile) and Canchayllo (Peru).

CHILE			PERU		
Case-households*	Control-households^§^	p-value (Fisher’s Exact test)	Case households*	Control households^§^	p-value (Fisher’s Exact test)
**DOGS’ FECES AND RECTAL SWABS **n PCR+ samples/N samples collected (% PCR+ samples)					
2/4 (50.0%)	2/37 (5.4%)	**0.041**	0/9 (0.0%)	0/24 (0.0%)	>0.900
**SOIL **n PCR+ samples/N samples collected (% PCR+ samples)					
2/9 (22.2%)	6/32 (18.8%)	>0.900	5/12 (41.7%)	3/33 (29.1%)	**0.022**
**SHOES’ SOLES **n PCR+ samples/N samples collected (% PCR+ samples)					
1/20 (5.0%)	13/58 (22.4%)	0.100	1/10 (10%)	10/30 (33.3%)	0.200
**DOG’S FUR **n PCR+ samples/N samples collected (% PCR+ samples)					
3/10 (30.0%)	21/72 (29.2%)	>0.90	2/14 (14.3%)	5/52 (9.6%)	0.600

Statistically significant differences upon Fisher’s Exact test are highlighted in bold. ^*^ Case-households are those households where at least one inhabitant with active abdominal CE was diagnosed by ultrasound screening. ^**§**^ Control-households are those households where no CE cases were identified by ultrasound screening.

In both countries, however, when applying a Bayesian logistic multilevel mixed-effects model (Table 2) there was no difference in risk of contamination according to location (“case households” and “control households”) nor according to matrix. Just in Peru the risk of dogs’ faeces and rectal swabs contamination was lower than the category of reference (soil), likely because no PCR positivity was detected in this matrix in Peru.

**Table 2:** Factors associated with PCR positivity for *Echinococcus granulosus* DNA in matrices collected at household’s level in Tulahuen (Chile) and Canchayllo (Peru) using Bayesian logistic multilevel mixed-effects model.

	CHILE		PERU	
	OR	95% CI	OR	95% CI
**Location**				
**Case household**	-		-	
**Control household**	1.17	0.31-4.52	0.67	0.22-2.07
**Matrix**				
**Soil**	-		-	
**Faeces and rectal swabs**	0.48	0.11-1.89	**0.00**	**0.00-0.16**
**Dogs’ fur**	2.30	0.84-6.89	0.55	0.17-1.71
**Shoe soles**	0.95	0.33-2.78	1.85	0.63-5.51

Statistically significant differences with the category of reference (-), evidenced by the absence of the value 1.00 within the 95% CI interval, are highlighted in bold. OR, odds ratio; CI, credible interval.

In Tulahuen (Chile) and Canchayllo (Peru), three public areas were identified for sampling. Contaminated faeces and soil samples were retrieved in all public areas, with the proportion of contamination ranging between 33–77% in faeces and 11–55% in soil in Tulahuen’s public areas, and between 12–50% in faeces and 11–55% in soil in Canchayllo. All vegetables collected from the two markets in Tulahuen and all flies collected in public areas in Canchayllo were PCR-negative for *E. granulosus* DNA. In both areas, matrices collected from public areas were more contaminated than matrices collected from households, which reached statistical significance by Fisher’s Exact test for both faecal and soil samples in Canchayllo and for faecal samples in Tulahuen (Table 3).

**Table 3:** Proportion of PCR positivity for *Echinococcus granulosus* DNA in matrices (soil and faecal material) collected at household’s level and in public areas (*e.g. village’s square) in Tulahuen (Chile) and Canchayllo (Peru).

CHILE				PERU			
Case households	Control households	Public areas*	p-value (Fisher’s Exact test)	Case households	Control households	Public areas*	p-value (Fisher’s Exact test)
**DOGS’ FECES OR RECTAL SWABS **n PCR+ samples/N samples collected (% PCR+ samples)							
2/4 (50.0%)	2/37 (5.4%)	15/25 (60%)	**<0.001**	0/9 (0.0%)	0/24 (0.0%)	6/18 (33.3%)	**0.001**
**SOIL **n PCR+ samples/N samples collected (% PCR+ samples)							
2/9 (22.2%)	6/32 (18.8%)	10/27 (37.0%)	0.300	5/12 (41.7%)	3/33 (9.1%)	8/17 (29.6%)	**0.023**

Statistically significant differences upon Fisher’s Exact test are highlighted in bold.

When applying a Bayesian logistic multilevel mixed-effects model (Table 4), in neither country analysed separately, or when the two countries were considered together, there was any difference in risk of contamination according to type of matrix. Control households were at significant lower risk of contamination in Peru and when the two countries were considered together, but not in Chile.

**Table 4:** Factors associated with PCR positivity for *E. granulosus* DNA in matrices (soil and faecal material) collected at household’s level and in public areas (* e.g. village’s squares) in Tulahuen (Chile) and Canchayllo (Peru), and the two areas together, using Bayesian logistic multilevel mixed-effects model.

	CHILE		PERU		CHILE AND PERU	
	OR	95% CI	OR	95% CI	OR	95% CI
**Location**						
**Case household**	-		-		-	
**Control household**	0.25	0.01-4.44	**0.11**	**0.01-0.89**	**0.19**	**0.04-0.84**
Public areas*	1.96	0.06-86.3	1.66	0.18-16.9	1.96	0.41-9.69
**Matrix**						
**Soil**	-		-		-	
**Faeces or rectal swabs**	2.45	0.91-6.92	0.44	0.14-1.35	0.87	0.45-1.70

Statistically significant differences with the category of reference (-), evidenced by the absence of the value 1.00 within the 95% CI interval, are highlighted in bold. OR, odds ratio; CI, credible interval.

## Discussion

Humans acquire *E. granulosus* infection by accidental ingestion of viable parasite eggs; however, very few studies examined different fomite’s contamination, which could be the source of such accidental ingestion. The vast majority of studies actually investigated dogs’ faecal samples collected from the environment, while contamination of soil, water and food received less attention, with soil generally found the most contaminated matrix ([12,27] and S1 Table). In this study, we evaluated *E. granulosus* contamination of matrices sampled from households and public areas in two villages of Chile and Peru highly endemic for CE, identified through abdominal US screening, applying a case-control study design. It can be assumed that (the majority of) parasite DNA derived from eggs present in the matrices and trapped in the 21 µm filter of the sequential sieving method applied to all matrices before molecular analysis.

Overall, our results show a generally high degree of contamination of households- and public areas-derived matrices from which hand-to-mouth ingestion of eggs could derive, including not only soil and, expectedly, faecal material, being especially contaminated in public areas and households, but also shoe soles and dogs’ fur. The level of contamination could have been even higher when considering factors limiting sensitivity of PCR (e.g., inhibitors in faeces). The observation of high levels of contamination of matrices collected in both public areas and “control households” is of particular importance since it highlights the general risk of infection which might derive from living in a contaminated environment, independently of having direct involvement with the parasite cycle (e.g., through owning dogs or livestock, home-slaughtering, etc.). Nematode eggs transfer through shoe soles was investigated previously [28]; here we demonstrated for the first time the carry-over of *E. granulosus* eggs through this fomite, expanding the evidence on ways of mechanical dispersal through waterfall, wind and animals [12]. No differences were found in proportions of contamination between locations inside the household (main house entrance, backyard’s entrance, dogs’ housing area), while the dogs’ perianal fur, followed by the muzzle area, were highly contaminated among matrices directly obtained from the dogs’ bodies. On the contrary, no contamination was found in leafy greens, either purchased or home-grown. It must be noted that the analysis of vegetables was possible only in Chile, in limited quantity, and we could not obtain information on their actual origin. Indeed, not many vegetables are grown in these areas because of the hard weather conditions. Taken together, these observations support the need for a paradigm shift from considering *E. granulosus* primarily a food- and water-borne infection, to more generally an environmental-borne infection, and highlight the risk deriving from living in an endemic area (community risk) as opposed to just a risk deriving from direct involvement in practices fostering in the transmission cycle of the parasite [13,29]. In accordance, the results of the risk factors questionnaire administered during the ultrasound screening in the same area of Chile [15] found increasing age and living/working in a rural setting being significantly associated with CE infection. These results are also consistent with the observation from population-based studies that CE prevalence increases with age, and that recent infections (arguably evidenced by the diagnosis of CE1 stage cysts) are observed in all age groups [15,17,30–32], reflecting the cumulative risk of infection to which inhabitants of endemic areas are exposed year after year. It is also reasonable to suppose that individual habits fostering hand-to-mouth contact with contaminated matrices should play a role in the individual risk of every person living in endemic areas [13,15]. Furthermore, different infection attributable fractions might derive from different sources in different areas, with food and non-food sources having different relative importance in different contexts, depending on the agricultural and consumption habits. For example, a recent multicentric study on *Echinococcus* spp. eggs contamination in berries and lettuces found very variable levels of contamination with *E. granulosus* eggs, with figures up to 12% in lettuces and a striking 81.3% in strawberry samples from one location in Tunisia [33]. This stresses the importance of integrating epidemiological studies with social sciences studies, aiming to identify those community-specific or individual-specific interventions that could effectively help preventing accidental ingestion of parasite eggs, as already undertaken for soil-transmitted helminths and taeniasis/cysticercosis, where the effectiveness of single components of WASH programs and the implementation of well-established health education methodologies are being investigated to improve control recommendations [13]. Such studies would also be pivotal to reach an evidence-based estimate of attributable fractions for infection, so far only derived from risk factors questionnaires or expert elicitation [14,34–36].

This study had several limitations. First, it is possible that some “control households” could have been erroneously classified as such, and instead having CE-positive household’s members. This is because we could not evaluate by US (and chest X ray to detect lung CE) all household members in all households. Regarding lung CE, however, it must be considered that this is less frequent than hepatic CE, and more often symptomatic [9], therefore it would constitute a lower proportion of asymptomatic CE in an apparently healthy population.

Second, we assessed contamination by *E. granulosus* eggs through detection of DNA obtained after filtration of matrices, without a microscopy step to visualize the actual eggs. Although, as mentioned above, it can be assumed that the majority of parasite DNA actually derived from eggs present in the matrices and trapped in the 21 µm filter, we cannot exclude that part of it could derive from other sources (e.g., parasite cells); however, this latter component is ought to be limited. A further implication is that the actual infectivity of the eggs in the matrices was not assessed and cannot be inferred. Eggs survival is prone to environmental conditions with heterogeneous survival rates, and eggs infectivity can be reduced by factors affecting their viability in the environment [12]. On the one hand, our retrieval of parasite DNA in superficial soil could reflect the general degree of environmental contamination, since eggs accumulate in the superficial layers of soil until degraded or washed away or stratified in lower layers from accumulation of new soil [12]. On the other hand, parasite contamination of faecal materials could more directly reflect an infectious matrix, since most of the DNA detected in faeces might come from infective eggs, which can survive for years [12] while faecal packs degrade in weeks. A further limitation concerning DNA detection is that in this study we did not carry out detection-limit experiments from the different matrices examined. In addition, the presence of PCR inhibitors in some matrices may have compromised DNA detection, potentially leading to an underestimation of the true prevalence.

Third, no direct causality can be of course inferred between CE infections diagnosed in the study participants and contamination detected in the actual matrices obtained from the environment where they live, due to the long and uncertain time intercurrent between the infection event (which is asymptomatic) and development (and then detection) of a CE cyst. We assumed that the general conditions of the studied places and therefore people’s exposure to infection did not change over the past years, and therefore the results obtained could photograph the epidemiological status of the investigated areas of present and near-past periods. However, we did not directly inquire about time of residence if the house facilities and changes in the household organization; for example, dog’s turnover in the households could be relatively high.

Finally, due to logistical constraints, the type and number of samples of each matrix that could be retrieved from each site and place within site were limited. The availability of a limited number of samples and the high proportion of negative PCR results, limited the possibility to precisely estimate and compare the risk of contamination. Furthermore, we did not investigate a number of matrices which could be the source of infection, such as people’s hands, or drinking water. Of note, the results of the risk factors questionnaire administered during the ultrasound screening in the same area of Chile found a higher risk of CE in people declaring consumption of non-potable water [15]; therefore, the actual contamination of this source should be investigated further. However, for both these samples, a likely very large sample size would be needed, due to the faster turnover of these matrices (through washing hands and flowing of water used for human consumption) compared to the more “static” matrices investigated in this study. In addition, other matrices such as vegetables and flies could not be thoroughly investigated in this study due to the unavailability of the actual samples from a number of sites. However, none of such samples that could be examined was positive for parasite DNA. This is in accordance with the results of the risk factors questionnaire administered in the same area of Chile, which found no risk associated with the consumption of raw vegetables [15]. On the same note, we found a considerable proportion of *E. granulosus* DNA contamination on dogs’ fur, but “touching dogs” was not associated with higher risk of CE in the questionnaire [15], and “contact with dogs” is found inconstantly as a risk factor for CE in the literature [29]. These results highlight the importance of more accurately studying the real role and attributable fraction of fresh vegetables consumption and transfer from dogs’ fur for CE infection in communities with different food and social habits, to convey targeted primary prevention messages [13].

To conclude, the results of *E. granulosus* DNA contamination of households- and public locations-derived matrices in two villages of Chile and Peru highly endemic for CE point towards the need for a paradigm-shift from considering CE primarily a food-borne infection to more generally an environmental-borne infection with a “community risk” to which people living in endemic areas area exposed. These results stress the need to deepen our biological knowledge of *E. granulosus* contamination of matrices and sociological knowledge of habits and customs fostering the individuals’ risk of infection specific for each endemic area.

## Supporting information

S1 Checklist STROBE (Strengthening the Reporting of Observational Studies in Epidemiology) checklist. (DOCX)

S1 Dataset Original data of matrices sampling and *E. granulosus* PCR results. (XLSX)

S1 Table Review of original papers investigating *Echinococcus granulosus* contamination, at species level, in environmental matrices in *E. granulosus* endemic areas. (DOCX)
