# Supplementary material for: Community risk of environmental-borne cystic echinococcosis transmission in South America: Results from the multistep cross-sectional and case-control PERITAS study
Source: PLoS Negl Trop Dis. 2025 Aug 6;19(8):e0013382. doi: 10.1371/journal.pntd.0013382 (PMC12342264; doi:10.1371/journal.pntd.0013382)
Supplement: S1 Checklist — (DOCX) [file pntd.0013382.s001.docx]

**S1 Checklist**. STROBE (Strengthening the Reporting of Observational Studies in Epidemiology) checklist.

|  | Item No | Recommendation | Page  No |
| --- | --- | --- | --- |
| **Title and abstract** | 1 | (*a*) Indicate the study’s design with a commonly used term in the title or the abstract**: the study title includes the description “cross-sectional and case-control study”** | 1 |
|  |  | (*b*) Provide in the abstract an informative and balanced summary of what was done and what was found: **Abstract includes methodology and principal findings with figures in %** | 2 |
| Introduction | | | |
| Background/rationale | 2 | Explain the scientific background and rationale for the investigation being reported. **The 4^th^ paragraph of the introduction illustrates the scant knowledge about parasite eggs contamination in matrices different from soil and dogs’ feces, the previous data suggesting that environmental origin of infection (not only food/waterborne) could be dominant, and the rational for conducting environmental contamination studies being the need to provide health messages targeted on the real source of infection rather than genernc ones** | 3-6 |
| Objectives | 3 | State specific objectives, including any prespecified hypotheses: **In this study, we aimed to evaluate *E. granulosus* contamination of households- and public locations-derived matrices in two highly endemic areas of Chile and Peru, identified through ultrasound-based CE screening studies performed during the project PERITAS** | 6 |
| Methods | | | |
| Study design | 4 | Present key elements of study design early in the paper: **PERITAS was conceived as a stepwise multidisciplinary study involving three stages (Figure 1). Stage 1 included an ultrasound population-based screening […], aiming to estimate the prevalence of human CE and to identify areas/villages with both households (with and without CE cases) and public areas […] where to carry out the environmental sampling. Stage 2 was a case-control study encompassing sampling of different matrices in the areas selected in stage 1 […]. Stage 3 aimed to detect E. granulosus s. l. DNA in the sampled matrices, to evaluate and compare contamination between areas.** | 7 |
| Setting | 5 | Describe the setting, locations, and relevant dates, including periods of recruitment, exposure, follow-up, and data collection: **Details of each study stage are presented in terms of setting, locations, dates, and sample and data collection** | 8-11 |
| Participants | 6 | (*a*) *Cohort study*—Give the eligibility criteria, and the sources and methods of selection of participants. Describe methods of follow-up **NA**  *Case-control study*—Give the eligibility criteria, and the sources and methods of case ascertainment and control selection. Give the rationale for the choice of cases and controls **The village where to carry out the environmental sampling was identified based on the presence of high prevalence of CE with active cyst stages in the ultrasound surveys and the presence of at least five households with participants with abdominal active CE. In each selected area, five households with at least one CE case with active cysts detected in the ultrasound surveys (“case households”) and 15 (1:3 ratio) households with no CE cases in the ultrasound surveys (“control households”) were selected for matrices sampling. Care was taken to select households with similar environmental conditions […] and, whenever possible, where all the family was screened by ultrasound**.  *Cross-sectional study*—Give the eligibility criteria, and the sources and methods of selection of participants **Stage 1 is briefly described in page 8 since it is published in PLoS NTD 2022 (ref 15 of the manuscript)** | 8-10 |
|  |  | (*b*) *Cohort study*—For matched studies, give matching criteria and number of exposed and unexposed **NA**  *Case-control study*—For matched studies, give matching criteria and the number of controls per case **In each selected area, five households with at least one CE case with active cysts detected in the ultrasound surveys (“case households”) and 15 (1:3 ratio) households with no CE cases in the ultrasound surveys (“control households”) were selected for matrices sampling** | 9 |
| Variables | 7 | Clearly define all outcomes, exposures, predictors, potential confounders, and effect modifiers. Give diagnostic criteria, if applicable. **For Stage 1, ultrasound diagnostic criteria are those of the WHO-IWGE; for Stage 2, variables (% contamination of matrices as assessed in stage 3) were shoe soles, owned dogs feces/rectal swabs, owned dogs’ fur, soil, and leafy greens for households and soil and dogs’ feces collected from the environment for common areas** | 8-10 |
| Data sources/ measurement | 8* | For each variable of interest, give sources of data and details of methods of assessment (measurement). Describe comparability of assessment methods if there is more than one group **Stage 3 describes molecular detection in matrices in matrices including sampling (avoiding cross-contamination). It is specified that molecular analyses were centralized in Chile to decrease inter-laboratory variability.** | 10-11 |
| Bias | 9 | Describe any efforts to address potential sources of bias **Stage 3 describes molecular detection in matrices in matrices including sampling (avoiding cross-contamination). It is specified that molecular analyses were centralized in Chile to decrease inter-laboratory variability.** | 10-11 |
| Study size | 10 | Explain how the study size was arrived at **Due to the absence of previous data on contamination of environmental matrices by E. granulosus eggs, sample size calculations were based on estimation of prevalence of active abdominal CE cysts in the investigated villages for Stage 1 [ref 15]. Based on a desired precision of 1% and a confidence level of 95%, and a maximum expected prevalence of active abdominal CE of 4% (maximum expected prevalence of abdominal CE 8%), the minimum US screening sample size was n= 374 in villages of 500 inhabitants, n=597 in villages of 1000 inhabitants, and n=850 in villages of 2000 inhabitants. For stage 2 and 3, the sample size was based on available resources and capacity.** | 11 |
| Quantitative variables | 11 | Explain how quantitative variables were handled in the analyses. If applicable, describe which groupings were chosen and why. **Households classification: households with at least one CE case with active cysts detected in the ultrasound surveys = “case households”; households with no CE cases in the ultrasound surveys = “control households”. PCR results of matrices were reported as positive or negative. Continuous variables were summarized using median and interquartile ranges (IQR), while discrete variables were summarized by absolute and percentage frequencies.** | 9,11,12 |
| Statistical methods | 12 | (*a*) Describe all statistical methods, including those used to control for confounding. **Frequencies were compared using Fisher's exact test. To identify the factors associated with contamination (i.e. location and matrices), we used a multilevel mixed-effects logistic regression analysis within a Bayesian framework, treating household/public area codes as the random effect. For the regression coefficients, we applied minimally informative priors following a normal distribution, while the variance was estimated using a Student-t (3, 0, 2.5) distribution.** W**e assessed convergence by tracking the Rhat statistic and monitored effective sample sizes using both Bulk ESS and Tail ESS** | 11-12 |
|  |  | (*b*) Describe any methods used to examine subgroups and interactions  **The McNemar test was used to compare the frequency of contamination between dogs’ muzzle fur, perianal fur, and rectal swabs/feces** | 11 |
|  |  | (*c*) Explain how missing data were addressed **NA** |  |
|  |  | (*d*) *Cohort study*—If applicable, explain how loss to follow-up was addressed **NA**  *Case-control study*—If applicable, explain how matching of cases and controls was addressed. **In each selected area, five households with at least one CE case with active cysts detected in the ultrasound surveys (“case households”) and 15 (1:3 ratio) households with no CE cases in the ultrasound surveys (“control households”) were selected for matrices sampling. Care was taken to select households with similar environmental conditions […] and, whenever possible, where all the family was screened by ultrasound**  *Cross-sectional study*—**If applicable, describe analytical methods taking account of sampling strategy. Continuous variables were summarized using median and interquartile ranges (IQR), while discrete variables were summarized by absolute and percentage frequencies**. **CE prevalence on ultrasound is published in PLoS NTD 2022 (ref 15 of the manuscript)** | 8-11 |
|  |  | (*e*) Describe any sensitivity analyses **NA** |  |

Continued on next page

| Results | | | |
| --- | --- | --- | --- |
| Participants | 13* | (a) Report numbers of individuals at each stage of study—eg numbers potentially eligible, examined for eligibility, confirmed eligible, included in the study, completing follow-up, and analysed. **In Chile, a total of 2,439 people were screened by ultrasound from 13 localities in Limarí province, Coquimbo region, finding a 1.6% mean prevalence of CE, with 51% of CE cysts being in active stages [15]. In Peru, a total of 1,181 people screened by ultrasound from 12 localities in Junin and Huancavelica regions, finding a 3.7% mean prevalence of CE, with 56% of CE cysts being in active stages (unpublished). In Chile, matrices sampling was carried out in Tulahuen, a rural area of 935 inhabitants in the municipality of Monte Patria of the Limarí province, Coquimbo region [15]. In this area, the ultrasound-based screening performed in 2019 found a CE prevalence of 4.5%, with 2.5% of participants having CE in active stages [15]. In Peru matrices were collected from Canchayllo, a rural village located in Junin region, in the Peruvian Central Highlands. Of the 768 inhabitants of Canchayllo (data from Instituto Nacional de Estadistica e Informatica), 236 were evaluated by ultrasound in this study. A prevalence of CE of 9,3% was found, and 50% of them had at least one active cyst. For matrices samples refer to the original data are available in S1 dataset.** | 12-14 |
|  |  | (b) Give reasons for non-participation at each stage **NA** |  |
|  |  | (c) Consider use of a flow diagram. **For matrices samples refer to the original data are available in S1 dataset.** |  |
| Descriptive data | 14* | (a) Give characteristics of study participants (eg demographic, clinical, social) and information on exposures and potential confounders. **This study refers to environmental matrices, not people. Details of the households’ occupants are presented in page 13: In Chile, the median number of household’s members were 3 (range 2-6) in the “control households” and 2 (range 2-5) in the “case households”. The number of family members having received abdominal ultrasound was 2 (range 1-3) in the “control houses” and 2 (range 2-2) in the “case households”. In Peru, five “case households” and 15 “control households” could be selected. The median number of household’s members were 3 (range 1-6) in the “control households” and 4 (range 2-7) in the “case households”; the number of family members having received abdominal ultrasound was 2 (range 1-4) in the “control houses” and 2 (range 1-5) in the “case households”.** | 13 |
|  |  | (b) Indicate number of participants with missing data for each variable of interest **NA** |  |
|  |  | (c) *Cohort study*—Summarise follow-up time (eg, average and total amount) **NA** |  |
| Outcome data | 15* | *Cohort study*—Report numbers of outcome events or summary measures over time **NA** |  |
|  |  | *Case-control study—*Report numbers in each exposure category, or summary measures of exposure **Complete results are presented in Table 1, 2, 3, and 4** | 16-19 |
|  |  | *Cross-sectional study—*Report numbers of outcome events or summary measures |  |
| Main results | 16 | (*a*) Give unadjusted estimates and, if applicable, confounder-adjusted estimates and their precision (eg, 95% confidence interval). Make clear which confounders were adjusted for and why they were included. **Stage 1: In Chile in the area selected for matrices sampling the ultrasound-based screening performed in 2019 found a CE prevalence of 4.5%, with 2.5% of participants having CE in active stages [15]. In Peru, a prevalence of CE of 9,3% was found, and 50% of them had at least one active cyst (unpublished)** | 12-13 |
|  |  | (*b*) Report category boundaries when continuous variables were categorized **NA** |  |
|  |  | (*c*) If relevant, consider translating estimates of relative risk into absolute risk for a meaningful time period **NA** |  |
| Other analyses | 17 | Report other analyses done—eg analyses of subgroups and interactions, and sensitivity analyses **NA** |  |
| Discussion | | | |
| Key results | 18 | Summarise key results with reference to study objectives**. Overall, our results show a generally high degree of contamination of households- and public areas-derived matrices from which hand-to-mouth ingestion of eggs could derive, including not only soil and, expectedly, faecal material, being especially contaminated in public areas and households, but also shoe soles and dogs’ fur. The observation of high levels of contamination of matrices collected in both public areas and “control households” is of particular importance since it highlights the general risk of infection which might derive from living in a contaminated environment, independently of having direct involvement with the parasite cycle (e.g. through owning dogs or livestock, home-slaughtering, etc.).** | 20 |
| Limitations | 19 | Discuss limitations of the study, taking into account sources of potential bias or imprecision. Discuss both direction and magnitude of any potential bias. **First, it is possible that some “control households” could have been erroneously classified as such, and instead having CE-positive household’s members**. **Second, we assessed contamination by E. granulosus eggs through detection of DNA obtained after filtration of matrices, without a microscopy step to visualize the actual eggs**. **A further limitation concerning DNA detection is that in this study we did not carry out detection-limit experiments from the different matrices examined. Third, no direct causality can be of course inferred between CE infections diagnosed in the study participants and contamination detected in the actual matrices obtained from the environment where they live. Finally, due to logistical constraints, the type and number of samples of each matrix that could be retrieved from each site and place within site were limited.** | 21-23 |
| Interpretation | 20 | Give a cautious overall interpretation of results considering objectives, limitations, multiplicity of analyses, results from similar studies, and other relevant evidence. **The results of E. granulosus DNA contamination of households- and public locations-derived matrices in two villages of Chile and Peru highly endemic for CE point towards the need for a paradigm-shift from considering CE primarily a food-borne infection to more generally an environmental-borne infection with a “community risk” to which people living in endemic areas area exposed**. | 24 |
| Generalisability | 21 | Discuss the generalisability (external validity) of the study results. **These results stress the need to deepen our biological knowledge of E. granulosus contamination of matrices and sociological knowledge of habits and customs fostering the individuals’ risk of infection specific for each endemic area.** | 24 |
| Other information | | | |
| Funding | 22 | Give the source of funding and the role of the funders for the present study and, if applicable, for the original study on which the present article is based. **This work was funded by EU-LAC Health (EULAC/FONIS T020067), through the PERITAS (Molecular epidemiological studies on pathways of transmission and long-lasting capacity building to prevent cystic echinococcosis infection) project (EULACH16/T02-0067). FT and CM are supported by funding from the Italian Ministry of Health “Ricerca Corrente”–L2. The funding sources had no role in study design, data collection and analysis, decision to publish, or preparation of the manuscript** | Online submission platform |

*Give information separately for cases and controls in case-control studies and, if applicable, for exposed and unexposed groups in cohort and cross-sectional studies.

**Note:** An Explanation and Elaboration article discusses each checklist item and gives methodological background and published examples of transparent reporting. The STROBE checklist is best used in conjunction with this article (freely available on the Web sites of PLoS Medicine at http://www.plosmedicine.org/, Annals of Internal Medicine at http://www.annals.org/, and Epidemiology at http://www.epidem.com/). Information on the STROBE Initiative is available at www.strobe-statement.org.
