# Supplementary material for: Community risk of environmental-borne cystic echinococcosis transmission in South America: Results from the multistep cross-sectional and case-control PERITAS study
Source: PLoS Negl Trop Dis. 2025 Aug 6;19(8):e0013382. doi: 10.1371/journal.pntd.0013382 (PMC12342264; doi:10.1371/journal.pntd.0013382)
Supplement: S1 Table — (DOCX) [file pntd.0013382.s003.docx]

**S1 Table**. Review of original papers investigating *Echinococcus granulosus* contamination, at species level, in environmental matrices in *E. granulosus* endemic areas.

The PubMed search [(echinococc*) AND (contamin*)] was carried out before the study (Jan 7, 2019) and updated before data analysis (Oct 4, 2024), with no restrictions on language or year of publication. The available results of an European study (MEMe) about contamination of vegetables from 5 European, Tunisia and Pakistan, in “submitted” status at the time of the PubMed search, were also included. A total of 22 papers were therefore included, 17 (77%) analysing dogs’ feces collected from the ground, four (18%) soil, three (14%) water, and four (18%) vegetables. *E. granulosus* was identified by PCR in 17 (77%) papers, while copro-ELISA was used in four (18%) and an immunofluorescence technique in one (4%) paper assessing water and soil. The rate of *E. granulousus* positivity in dogs’ feces ranged between 0-32%, soil between 0-25% (0-8% if considering only PCR-based detections); water between 0-60% (0-2% if considering only PCR-based detections); and green vegetables between 0-12%. The studies were carried out in Africa (n=9), Asia/Middle east (n=8), Europe (n=4), and South America (n=3). No study investigated E*. granulosus* contamination of environmental matrices other than dispersed dogs’ feces in South America.

| **First Author, year** | **Country** | **Matrices** | **Technique** | **Sample size** | **% *E. granulosus s.l.* positivity** |
| --- | --- | --- | --- | --- | --- |
| Umhang G et al., 2025 [1] | Europe | Vegetables | PCR after filtration | 695 | Mean 1.3% |
|  | Tunisia |  |  | 75 | 12% |
|  | Pakistan |  |  | 100 | 4% |
|  | Europe | Strawberries |  | 199 | Mean 1.5% |
|  | Tunisia |  |  | 16 | 81.3% |
|  | Pakistan |  |  | - | - |
|  | Europe | Blueberries |  | 79 | Mean 1.3% |
|  | Tunisia |  |  | - | - |
|  | Pakistan |  |  | 25 | 12.0% |
| Shamsaddini S et al., 2024 [2] | Iran | Dispersed dogs’ feces | PCR after flotation | 5607 | 4.8% |
| Abdykerimov KK et al., 2024 [3] | Kyrgyzstan | Dispersed dogs’ feces | PCR after flotation | 2013 | 3.5%-4.2% |
| Ritossa L et al ., 2023 [4] | Argentina | Dispersed dogs’ feces | *E.g.* copro-ELISA | 360 | 11.3% |
| Awosanya EJ et al., 2022 [5] | Nigeria | Soil | PCR after flotation | 200 | 8% |
|  |  | Dispersed dogs’ feces | PCR after flotation | 200 | 24% |
|  |  | Water | PCR | 50 | 2% |
| Zhang X et al., 2022 [6] | China | Dispersed dogs’ feces | PCR from feces | 682 | 0% |
| Serra E et al., 2022 [7] | Italy | Water | PCR after filtration | 35 | 0% |
|  |  | Soil | PCR after flotation | 33 | 0% |
|  |  | Vegetables | PCR after filtration | 23 | 0% |
|  |  | Dispersed dogs’ feces | PCR after flotation | 25 | 32% |
| M'rad S et al., 2021 [8] | Tunisia | Dispersed dogs’ feces | PCR | 288 | 11.1% |
| Sharma PM et al., 2021 [9] | Bhutan | Dispersed dogs’ feces | PCR after flotation | 953 | 3.7% |
| M'rad S et al., 2020 [10] | Tunisia | Vegetables | PCR after flotation | 240 | 1.2% |
| Mulinge E et al., 2018 [11] | Kenya | Dispersed dogs’ feces | PCR after flotation | 1621 | 4.4% |
| Flores V et al., 2017 [12] | Argentina | Dispersed dogs’ feces | *E.g.* Copro-ELISA | 188 | 9.3% |
| Federer K et al., 2016 [13] | Switzerland | Vegetables | PCR after filtration | 46 | 4.3% |
| Niu YL et al., 2016 [14] | Tibet | Dispersed dogs’ feces | *E.g.* copro-ELISA | 226 | 23.9% |
| Chaâbane-Banaoues R et al., 2016 [15] | Tunisia | Dispersed dogs’ feces | PCR | 553 | 24.9% |
| Chaâbane-Banaoues R et al., 2015 [16] | Tunisia | Dispersed dogs’ feces | PCR after flotation | 1095 | 25.3% |
| Laurimaa L et al., 2015 [17] | Estonia | Dispersed dogs’ feces | PCR from feces | 90 | 2.2% |
| Mateus TL et al., 2014 [18] | Portugal | Dispersed dogs’ feces | PCR after flotation | 296 | 0% |
| Van Kesteren F et al., 2013 [19] | Kyrgyzstan | Dispersed dogs’ feces | PCR from feces | 104 | 3.8% |
| Acosta-Jamett G et al., 2010 [20] | Chile | Dispersed dogs’ feces | *E.g.* copro-ELISA | 334 | 7.2% |
| Shaikenov BS et al, 2004 [21] | Kazakhstan | Soil | PCR | 120 | 4.2% |
| Craig PS et al, 1988 [22] | Kenya | Water | Immunostaining after eggs hatching | 5 | 60% |
|  |  | Soil |  | 32 | 25% |

1. Umhang G, Bastien F, Cartet A, Ahmad H, van der Ark K, Berg R, et al. Detection of *Echinococcus spp*. and other taeniid species in lettuces and berries: Two international multicenter studies from the MEmE project. Int J Food Microbiol. 2025;430:111059
2. Shamsaddini S, Schneider C, Dumendiak S, Aghassi H, Kamyabi H, Akhlaghi E et al. Environmental contamination with feces of free-roaming dogs and the risk of transmission of *Echinococcus* and *Taenia* species in urban regions of southeastern Iran. Parasit Vectors. 2024;17:359.
3. Abdykerimov KK, Kronenberg PA, Isaev M, Paternoster G, Deplazes P, Torgerson PR. Environmental distribution of *Echinococcus*- and *Taenia spp*.-contaminated dog feces in Kyrgyzstan. Parasitology. 2024;151:84-92.
4. Ritossa L, Viozzi G, Lazzarini L, Pierangeli N, Flores V. Canine parasitoses in north Patagonia (Argentina): comparison between different social and environmental factors. J Helminthol. 2023;97:e77.
5. Awosanya EJ, Olagbaju A, Peruzzu A, Masu G, Masala G, Bonelli P. Detection of *Echinococcus granulosus sensu lato* in environmental samples from Ibadan, Oyo State, South West Nigeria. Vet Sci. 2022;9:679.
6. Zhang X, Jian Y, Ma Y, Li Z, Fu Y, Cairang Z et al. Prevalence of intestinal parasites in dog faecal samples from public environments in Qinghai Province, China. Pathogens. 2022;11:1240.
7. Serra E, Masu G, Chisu V, Cappai S, Masala G, Loi F et al. Environmental contamination by *Echinococcus spp*. eggs as a risk for human health in educational farms of Sardinia, Italy. Vet Sci. 2022;9:143.
8. M'rad S, Chaâbane-Banaoues R, Ghrab M, Babba H, Oudni-M'rad M. Human and animal cystic echinococcosis in Tataouine governorate: hypoendemic area in a hyperendemic country, myth or reality? Parasit Vectors. 2021;14:216.
9. Sharma PM, Thapa NK, Tshomo P, Dema T, Alvarez Rojas CA, Tenzin T et al. Occurrence of *Echinococcus granulosus sensu lato* and other taeniids in Bhutan. Pathogens. 2021;10:330.
10. M'rad S, Chaabane-Banaoues R, Lahmar I, Oumaima H, Mezhoud H, Babba H, et al. Parasitological contamination of vegetables sold in Tunisian retail markets with helminth eggs and protozoan cysts. J Food Prot. 2020;83:1104-1109.
11. Mulinge E, Magambo J, Odongo D, Njenga S, Zeyhle E, Mbae C et al. Molecular characterization of *Echinococcus* species in dogs from four regions of Kenya. Vet Parasitol. 2018;255:49-57.
12. Flores V, Viozzi G, Garibotti G, Zacharias D, Debiaggi MF, Kabaradjian S. Echinococcosis and other parasitic infections in domestic dogs from urban areas of an Argentinean Patagonian city. Medicina (B Aires). 2017;77:469-474.
13. Federer K, Armua-Fernandez MT, Gori F, Hoby S, Wenker C, Deplazes P. Detection of taeniid (*Taenia spp., Echinococcus spp*.) eggs contaminating vegetables and fruits sold in European markets and the risk for metacestode infections in captive primates. Int J Parasitol Parasites Wildl. 2016;5:249-253.
14. Niu YL, Wu WP, Guan YY, Wang LY, Han S, Gongsang QZ et al. Dog fecal contamination by *Echinococcus* in Cuomei County of Tibet in 2015. Zhongguo Ji Sheng Chong Xue Yu Ji Sheng Chong Bing Za Zhi. 2016;34:137-143
15. Chaâbane-Banaoues R, Oudni-M'rad M, M'rad S, Mezhoud H, Babba H. Environmental contamination by *Echinococcus granulosus sensu lato* eggs in relation to slaughterhouses in urban and rural areas in Tunisia. Korean J Parasitol. 2016;54:113-118.
16. Chaâbane-Banaoues R, Oudni-M'rad M, Cabaret J, M'rad S, Mezhoud H, Babba H. Infection of dogs with *Echinococcus granulosus*: causes and consequences in an hyperendemic area. Parasit Vectors. 2015;8:231.
17. Laurimaa L, Davison J, Süld K, Plumer L, Oja R, Moks E et al. First report of highly pathogenic *Echinococcus granulosus* genotype G1 in dogs in a European urban environment. Parasit Vectors. 2015;8:182.
18. Mateus TL, Castro A, Ribeiro JN, Vieira-Pinto M. Multiple zoonotic parasites identified in dog feces collected in Ponte de Lima, Portugal-a potential threat to human health. Int J Environ Res Public Health. 2014;11:9050-9067.
19. Van Kesteren F, Mastin A, Mytynova B, Ziadinov I, Boufana B, Torgerson PR et al. Dog ownership, dog behaviour and transmission of *Echinococcus spp*. in the Alay Valley, southern Kyrgyzstan. Parasitology. 2013;140:1674-1684.
20. Acosta-Jamett G, Cleaveland S, Bronsvoort BM, Cunningham AA, Bradshaw H, Craig PS. *Echinococcus granulosus* infection in domestic dogs in urban and rural areas of the Coquimbo region, north-central Chile. Vet Parasitol. 2010;169:117-122.
21. Shaikenov BS, Rysmukhambetova AT, Massenov B, Deplazes P, Mathis A, Torgerson PR. Short report: the use of a polymerase chain reaction to detect *Echinococcus granulosus* (G1 strain) eggs in soil samples. Am J Trop Med Hyg. 2004;71:441-443.
22. Craig PS, Macpherson CN, Watson-Jones DL, Nelson GS. Immunodetection of *Echinococcus* eggs from naturally infected dogs and from environmental contamination sites in settlements in Turkana, Kenya. Trans R Soc Trop Med Hyg. 1988;82:268-274.
